# Supplementary material for: Investigation of DNA Hybridization on Nano-Structured Plasmonic Surfaces for Identifying Nasopharyngeal Viruses
Source: Bioengineering (Basel). 2023 Oct 13;10(10):1189. doi: 10.3390/bioengineering10101189 (PMC10604513; doi:10.3390/bioengineering10101189)
Supplement: Supplementary file 1 [file bioengineering-10-01189-s001.zip › bioengineering-2624357-SI.pdf]

Supplemental Materials

Investigation of DNA Hybridization on Nano-Structured Plasmonic Surfaces for Identifying Nasopharyngeal Viruses

Shao-Sian Li [1,†], Yi-Jung Lu [2,†], Ray Chang [3], Ming-Han Tsai [4], Jo-Ning Hung [4], Wei-Hung Chen [3], Yu-Jui Fan [5,*], Pei-Kuen Wei [6,*] and Horn-Jiunn Sheen [3,*]

1. Department of Materials and Mineral Resources, National Taipei University of Technology, Taipei 10608, Taiwan; ssli@mail.ntut.edu.tw
2. Division of Family and Operative Dentistry, Department of Dentistry, Taipei Medical University Hospital, Taipei 11031, Taiwan; yi_jung2002@yahoo.com.tw
3. Institute of Applied Mechanics, National Taiwan University, No. 1, Section 4, Roosevelt Rd, Taipei 10617, Taiwan; d11543004@ntu.edu.tw (R.C.); r09543071@ntu.edu.tw (W.-H.C.)
4. Institute of Microbiology & Immunology, National Yang Ming Chiao Tung University. No. 155, Section 2, Linong St., Beitou District, Taipei 11221, Taiwan; m.tsai@gm.ym.edu.tw (M.-H.T.); cheeseerer0707@gm.ym.edu.tw (J.-N.H.)
5. School of Biomedical Engineering, College of Biomedical Engineering, Taipei Medical University, Taipei 11031, Taiwan
6. Research Center for Applied Sciences, Academia Sinica, 128 Academia Road, Section 2, Nankang, Taipei 11529, Taiwan

* Correspondence: ray.yj.fan@tmu.edu.tw (Y.-J.F.); pkwei@sinica.edu.tw (P.-K.W.); sheenh@ntu.edu.tw (H.-J.S.)

† These authors contributed equally to this work.

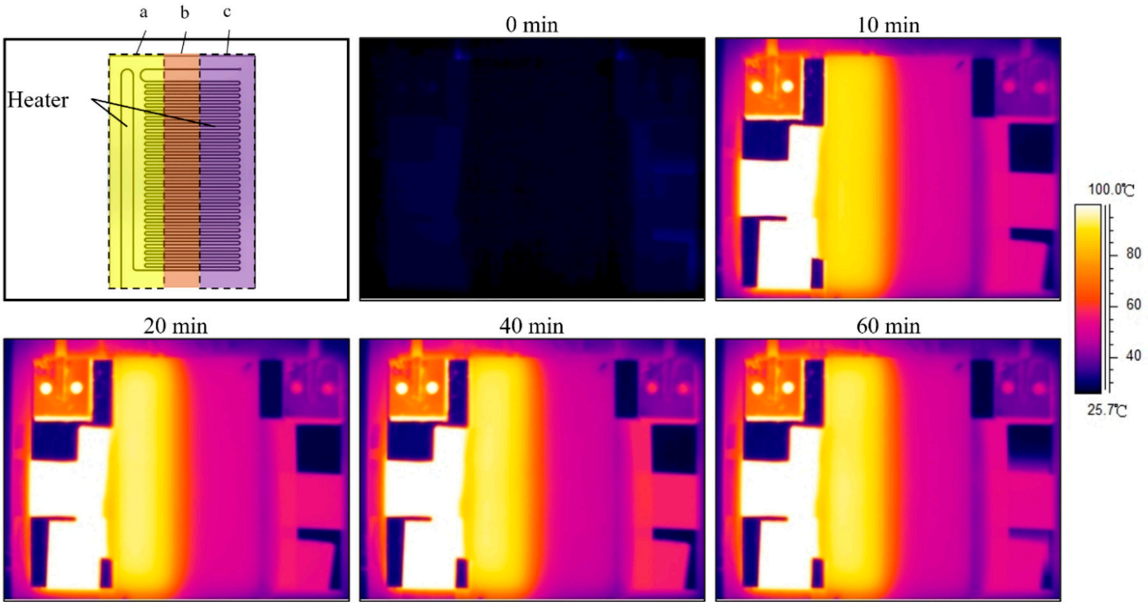

Figure S1. Characterization of the device. Temperatures were steady in the three regions; a (95 °C), b (65 °C), and c (50 °C). These temperature regions are used for denaturation (95 °C), extension (80-65 °C), detection (65 °C), and annealing (50 °C).

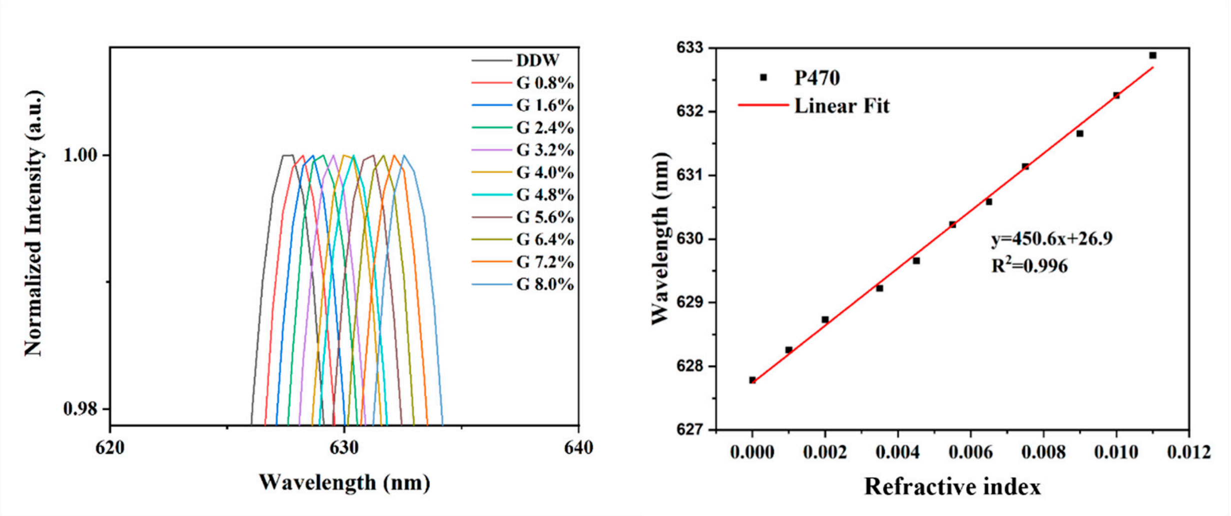

Figure S2. Relations between the resonance wavelength and refractive index in different glucose solutions.

Table S1. Primer templates and probes

	COVID-19 N-gene	*LMP1*
Template	5′-CACATTGGCACCCGCAATCCTGCTA<br>ACAATGCTGCAATCGTGCTACAACTT<br>CCTCAAGGAACAACATTGCCAAAAGG<br>CTTCTACGCAGAAGGGAGCAGAGGCG<br>GCAGTCAAGCCTCTTCTCGTTCCTC-3′	5′-AGCGACTCTGCTGGAAATGATGGA<br>GGCCCTCCAAAATTGACGGAAGAGGT<br>TGAAAACAAAGGAGGTGACCGGGGCC<br>CGCCTTCGATGACAGACGGTGGCGGC<br>GGTCATCCACACCTTCCTACACTGCTT<br>TTGGGTACTTCTGGTTCCGGTGGAGAT<br>GATGACGACCCCCACGGCCCAGTTCA<br>GCTAAGCTACTATGACTAACCTTTCTT<br>TACTTCTAGGCATTACCATGTCATAGG<br>CTTGCCTGACTGACTCTCCCTCCATTT<br>ACTGGGAATGCCTTAGCTAATCA<br>-3′
Forward	5′-CAC ATT GGC ACC CGC AAT C-3′	5′-AGC GAC TCT GCT GGA AAT GAT-3′
Reverse	5′-GAG GAA CGA GAA GAG GCT TG-3′	5′-TGA TTA GCT AAG GCA TTC CCA-3′
Probe	5′- TGG CAA TGT TGT TCC TTG AGG AAG T -3′	5′-GTC ATA GTA GCT TAG CTG AAC TGG GCC GT-3′
Probe's T_m	58.8 ℃	63.7 ℃

Table S2. Solution composition for PCR

	Volume	Final concentration
2X Master Mix	50 µl	1X
Primer F (10 µM)	2 µl	0.2 µM
Primer R (10 µM）	2 µl	0.2 µM
DNA template (10 ng/µL)	1 µl	-
Nuclease-free water	25 µl	-
**Total volume**	100 µl	-

Table S3. Solution composition for duplex PCR

	Volume	Final concentration
2X Master Mix	50 µl	1X
COVID-19 N-gene Primer F (10 µM)	2 µl	0.2 µM
COVID-19 N-gene Primer R (10 µM)	2 µl	0.2 µM
COVID-19 N-gene DNA template (10 ng/µL)	1 µl	-
LMP1_Primer F (10 µM)	2 µl	0.2 µM
LMP1_Primer R (10 µM)	2 µl	0.2 µM
LMP1 DNA template (10 ng/µL)	1 µl	-
Nuclease-free water	25 µl	-
**Total volume**	100 µl	-

Table S4. The conditions of the traditional PCR machine.

Initial denaturation	95 °C	5 min
Denaturation	95 °C	30 sec
Annealing	60 °C	50 sec
Extension	72 °C	50 sec
Final extension	72 °C	10 min
Storage	4 °C	10 min
